# The Perception of Primary School Teachers Regarding the Pharmacotherapy of Attention Deficit Hyperactivity Disorder

**DOI:** 10.3390/ijerph18126233

**Published:** 2021-06-09

**Authors:** João Gregório, Raquel Ferreira, Ana Sofia Fernandes

**Affiliations:** CBIOS, Research Center for Biosciences & Health Technologies, Universidade Lusófona’s, 1749-024 Lisboa, Portugal; raquel.lmf@live.com.pt (R.F.); ana.fernandes@ulusofona.pt (A.S.F.)

**Keywords:** attention deficit hyperactivity disorder (ADHD), pharmacotherapy, pharmacovigilance, school teachers

## Abstract

Attention Deficit Hyperactivity Disorder (ADHD) is raising concerns across health systems, affecting about 5% of the school-age population. Therapy usually involves psychostimulants, which are prone to adverse drug reactions (ADRs). Teachers have many contact hours with children and can easily detect behavioral changes upon the beginning of medication. However, few studies have focused on the role of teachers in the management of ADHD children and detection of ADRs. The present work aimed to characterize the perception of primary school teachers regarding the impact of ADHD therapeutics. A questionnaire was constructed focused on teachers’ training regarding ADHD and its therapy; experience with students with ADHD; changes upon beginning of medication; and observation of ADRs. A total of 107 completed questionnaires were obtained. The results indicate that more than 40% of the inquired teachers have received training in ADHD, but in most cases, the theme of therapeutics was absent from that training. The vast majority of teachers (91.6%) have had students with ADHD and observed mood alterations associated with medications. More than 60% of the teachers answered that they are aware of the ADRs and of these, 24% have already detected them in their students. The teachers reported the observed ADRs to parents in 93% of the cases and to doctors in 28% of the cases. In conclusion, the results show the need to reinforce teachers’ training in ADHD and its therapeutics.

## 1. Introduction

Attention Deficit Hyperactivity Disorder (ADHD) is a neurodevelopment disorder characterized by symptoms of hyperactivity, impulsivity, and inattention in excess for a person’s certain age or development stage [1,2]. Although the etiology of the disorder is not completely understood, many authors consider that it is a multidimensional disorder resulting from the interaction of genetic, environmental, psychological, and contextual factors [3,4,5].

The increasing diagnosis of ADHD in the last decades is concerning parents and teachers across countries [4,6]. This disorder affects about 5% of the school-age population, potentially threatening their mental and psychological development [1]. The diagnosis of ADHD should only be made by a specialist psychiatrist, paediatrician, or other appropriately qualified health professional. Apart from meeting the diagnostic criteria of the Diagnostic and Statistical Manual of Mental Disorders (DSM-5) or the International Classification of Diseases (ICD-10) on hyperkinetic disorder, all diagnostics must also include the assessment of a person’s needs, co-existing conditions, social, family, educational or occupational circumstances and physical health, and in the case of children, there must also be an assessment of the mental health of their parents or caregivers [2]. Although the ADHD persistent pattern of hyperactivity, impulsiveness, and lack of attention is typically diagnosed in early childhood, it is many times considered a chronic disease with symptom manifestation until adulthood [4]. Failing to treat ADHD is associated with low academic achievement, high unemployment, and use of illicit drugs and alcoholism. It is also associated with high rates of low social adjustment, marital and family conflict, and increased crime [7].

Recent guidelines in managing ADHD point to a comprehensive treatment plan encompassing both pharmacological and non-pharmacological approaches [1]. The most relevant therapeutic group prescribed are central nervous system stimulants (psychostimulants), with methylphenidate being the first line drug and the more commonly prescribed [8]. However, these drugs have several side-effects and adverse reactions (ADRs), ranging from insomnia and decreased appetite to headaches, drowsiness, sadness, and euphoria [9]. Although psychostimulants are amongst the drugs responsible for more adverse reactions in children populations [9], ADRs seem to go underreported [10].

Due to the early onset of the disease, its symptoms are often first detected by school teachers [11]. Children with ADHD have some characteristics that distinguish them from others who do not have the disorder. In school settings, they often do not follow rules, do not stay focused, especially on tasks that require longer mental effort, have difficulty answering questions and following directions even when they are directly addressed, do not sit still when necessary, have difficulty persisting with tasks and finishing them, and do not find it easy to preserve the information they are acquiring as they complete a task. As a consequence, these children have more problems relating to their peers and have more difficulty in keeping their friends, have low self-esteem and feel anxious, making it harder for them to do their schoolwork. Moreover, teachers are also the first to notice any change in child behavior, due to the high number of contact hours they have with students. Thus, they are well positioned to detect therapeutic response and ADRs of the prescribed medicines [12]. As such, it is essential to know what knowledge they have about this disorder, so that they can monitor their students with more information and awareness. However, only few studies have focused on this problem. Previous reports have documented that teachers usually have more knowledge about the diagnosis and most common symptoms of ADHD than about its treatment [13,14]. They also state that the more contact teachers have with these students, the more understanding about this disease they possess. These studies also suggest that the greater this contact, the more confidence teachers have. If they have more confidence, they are more successful in the pedagogical and behavioral management strategies they have to implement with the children [13]. On the other hand, a good relationship at family and school level has been found to improve the symptoms of the disease. It is therefore important that family and school relationships are of quality so that the symptoms of the disease are not aggravated, because these relationships can worsen or lessen the manifestations of the pathology [5].

Pharmacovigilance reporting systems are a precious asset in every health system. They provide the most data on ADRs. Nevertheless, their limitations are known, with under-reporting being one of the most common [15]. Legislation across Europe has adapted to include spontaneous reporting of ADRs aiming to the early detection of new or more serious ADRs [16]. ADRs spontaneous reporting systems are one of the World Health Organization’s (WHO) five minimum requirements for a functional national Pharmacovigilance system [17]. In these spontaneous reporting systems, healthcare professionals, patients, and their caregivers play a very active role [18]. Since 2002, ADRs reports from patients and consumers have increased exponentially [19]. Regarding ADHD drugs, the increase in prescriptions observed since 2010 has not been followed by an increase in ADRs reporting [20]. As the managing of ADHD behavior and therapeutic challenges becomes a team effort, shared by parents, teachers, and healthcare professionals [21], it is of great importance to study non-healthcare professionals’ knowledge and attitudes towards ADRs detection and notification. There is a dearth of studies focusing on the role teachers play in ADRs detection. Parents and teachers rating scales have long been developed to assess child behavior in drug studies [22], but none was specifically developed to assess teachers’ perceptions of the existence of a pharmacovigilance system and ADRs’ detection regarding ADHD.

Therefore, our aim was to explore primary school teachers’ perceptions about ADHD therapy efficacy and safety, as well as to understand their knowledge of the Portuguese pharmacovigilance system.

## 2. Materials and Methods

### 2.1. Development of the Data Collection Instrument

In order to accomplish the aim of this study, a survey had to be developed. We started with a literature review on the subject, performed in Pubmed and Google Scholar databases. Several papers of interest were found, focusing on drug therapy, ADHD diagnostic tools, ADRs rating scales, and teachers or parents’ role in the detection of ADRs [4,7,8,12,13,23].

Although many papers were found about the role of teachers in dealing with children with ADHD, none reported the use of an instrument to ADRs detection that could be applied to our population. As such, we decided to develop a set of questions inspired by the literature review that would allow the exploration of teachers’ perceptions on the subject. The survey consisted of 12 questions, including socio-demographic variables, open and closed questions about teachers’ perceptions of ADHD and its therapy, including perceived impact on children’s behavior; teachers’ education and training relative to ADHD therapy; number and type of ADRs identified and to whom they were reported.

The survey was then piloted in its paper version on a group of 12 teachers, representative of the study’s population. The possibility to convert this version to an online version was also assessed during this stage, to allow for maximum dissemination. After assessing the feasibility of this survey, an online version was designed, using Google^®^ Forms platform.

### 2.2. Study Population and Data Collection

The defined study population were all 1st–4th grade public school teachers (1st cycle or primary school) in the greater Lisbon area. Schools in Portugal are grouped according to a set of rules established by the Education Ministry, supported by demographic and geographic criteria [24]. 229 groups of schools constitute this region with 7805 primary school teachers. An email was sent to the directors of each group of schools, asking them to disseminate the online survey to all their first cycle teachers, after assessing the suitability of the survey to their setting. Data collection took place during two months, between 17 July and 15 September 2018.

### 2.3. Statistical Analyses

Descriptive statistics and all statistical analyses were performed using Microsoft Excel^®^ (Microsoft, Redmond, Washington, DC, USA) and SPSS^®^ v.22 (IBM Corp., Armonk, NY, USA). Chi-square tests were used to assess association between categorical variables. The significance level was set to 5%.

## 3. Results

The online survey yielded 107 responses, of which, 89.7% were women. The mean age of the sample was 47 years, with 68.2% being born before 1975. A minimum of 2 and a maximum of 44 years of teaching experience were reported, with an average of 21.6 years of teaching experience. 22% of teachers had less than 10 years of teaching experience, and 29.2% had more than 25 years of experience.

**Figure 1:**
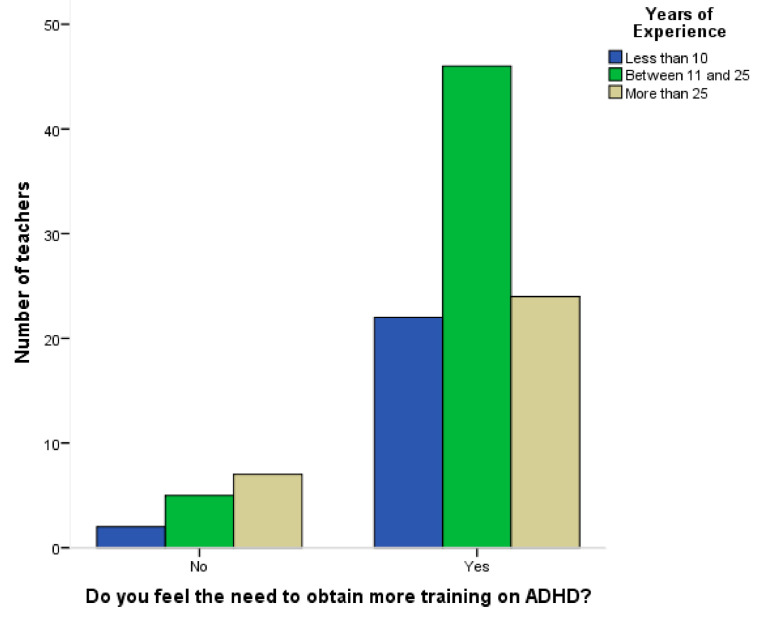
Perception of training needs according to years of experience.

In this sample, only 42.1% of the teachers reported to have had training on ADHD, and only 37.2% of the trained teachers stated that drug therapies’ subjects were included in their training. Nevertheless, 86.9% of teachers mention that they feel the need of training on this subject, with this need being independent of the years of experience (Chi-square 3.389; *p* = 0.184) (Figure 1).

**Figure 2:**
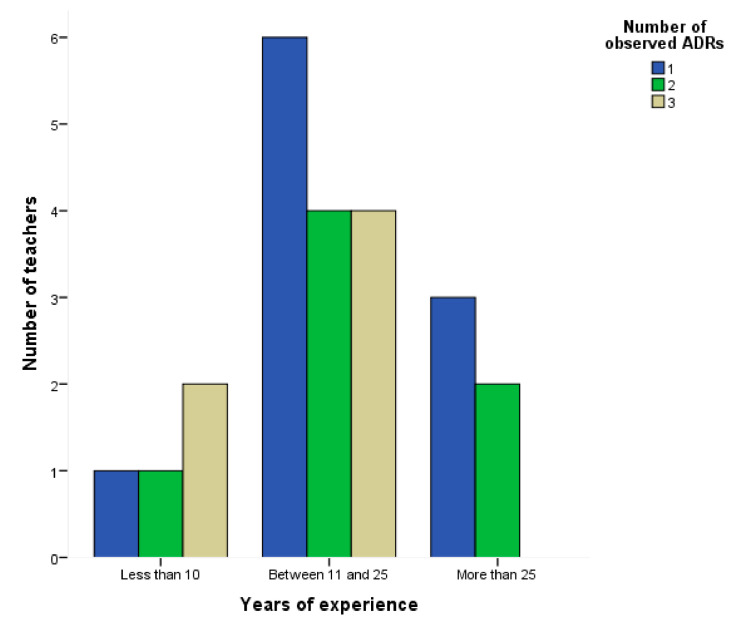
Number of observed adverse drug reactions by years of teaching experience.

The large majority of these teachers (91.6%; *n* = 99) answered that they already had students with ADHD, with 76 teachers declaring that they had students who were medicated after being in their class. 91.9% of teachers reported that the medication produced changes in behavior, most notably less agitated (87.6%), less impulsive (83.7%), and more attentive (76.2%) students. Regarding medication adverse reactions, 63.6% of teachers reported to be aware of ADRs but only 26% said that they found at least one ADR in their students. Twenty-seven teachers observed 42 possible ADRs, an average of 1.6 ADRs per teacher. Amongst these ADRs, apathy was the mostly cited (48.1% of teachers), followed by loss of appetite (25.9%), and stomach and abdominal problems (for example, abdominal pain, digestive problems, nausea). Several ADRs were cited only once, such as “weight gain”, “indifference”, “low self-esteem”, “anxiety, sadness and depression”, “absences”, and “prostration”. More experienced professionals had a tendency to detect more ADRs (Chi-square: 3.049; *p* = 0.550) (Figure 2).

**Figure 3:**
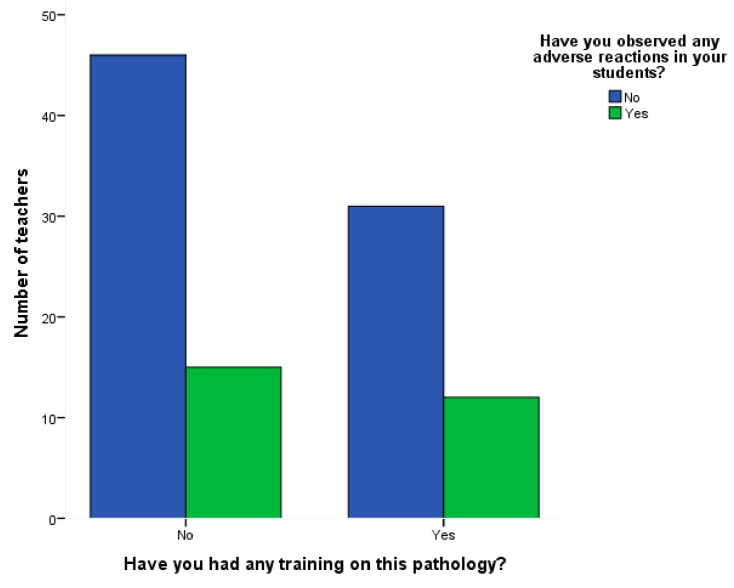
Observed adverse drug reactions by teachers with and without training on ADHD.

When considering the effect of teachers’ training about ADHD on adverse drug reaction detection, it was found that the effect of training did not affect the observation of ADRs (Chi-square: 0.144; *p* = 0.704) (Figure 3).

All the teachers that found ADRs in their students reported it to the parents. Only 29.2% reported it also to the child’s physician and none reported it to the National Pharmacovigilance System (NPS). When asked why they did not report, 72% (*n* = 18) stated that they are unaware of the NPS or of the process of reporting ADRs; while 20% said that they consider that ADRs reporting is not a teacher’s responsibility.

## 4. Discussion

According to the General Directorate for Statistics in Education and Science (DGEEC), 80% of primary school teachers are women, which is a similar proportion to that of our respondents. In addition, the average age of 47 years old indicates that our respondents may be representative of a typical first cycle teacher population in Lisbon region [24]. The respondents’ years of experience is indicative of well-established professionals that are experienced in dealing with usually heterogeneous classes.

Our results suggest that most teachers in greater Lisbon have had contact with at least one student with ADHD. In fact, it appears that almost all teachers have had contact with students with this condition and that approximately 50% consider that they show essentially beneficial changes with the medication. Most feel that students have become more attentive, less agitated, less distracted, less impulsive, and more emotionally stable. Since these are the symptoms associated with the disease that often lead to taking medications, it seems teachers’ perceptions are that the medications are efficient for the purposes for which they were developed. However, teachers tend to over-estimate the number of children with ADHD in their classes, and this estimate is amplified by classes’ size [11]. Our survey did not explore class size in relation to ADRs detection. As such, future studies can explore the relation with this additional parameter.

Although some teachers referred that they had training about ADHD, the large majority felt the need to have more training about this subject, preferably including medication-related themes. This may be the reason behind the non-apparent effect of training in ADRs detection: it seems that current training programs do not highlight the subject of ADRs and do not emphasize its importance to a well-managed ADHD patient. However, years of experience seem to be associated with a higher detection of ADRs, confirming the importance of experience and confidence in identifying and managing children with ADHD [14]. This hints that future training strategies must include modules about drug therapy, ADRs detection and reporting, and should also include teachers with first-hand experience of managing children with ADHD as trainers.

In this study, “apathy” was the adverse reaction most often perceived by teachers. Since “apathy” is not one of the most frequent ADRs in these medications, this fact seems to highlight that this symptom is the trigger for teachers to refer a child as having ADHD. However, “apathy” may be what in other studies was identified as “staring”. This “staring” side-effect has frequently been reported in some studies [12,25]. Confounding “apathy” with “staring” may explain why most teachers in our study refer this to be the most prevalent adverse reaction. “Weight gain” was also reported once. This sign is not described as a presumed adverse reaction. However, a possible explanation for it is that some of these patients experience a decreased appetite in the beginning of the therapy. Because they do not eat the same way, there is a delay in their rate of growth in relation to weight and height. However, after some time, the tendency is for the initial weight loss and growth delay to be reversed. Thus, there is no weight gain associated with taking the medication, but there is a reposition, after some time, of the weight initially lost [26]. In addition, signs of “indifference”, “isolation”, “low self-esteem”, “disinterest”, “anxiety, sadness and depression”, “absences”, and “prostration” individually are not described as being expected adverse reactions. However, when they are aggregated, they describe manifestations characteristic of depression, an adverse reaction described for some of these ADHD medications. It should also be noted that weight loss or gain and sleepiness may also be associated with depression, so each case must be analyzed individually [1].

When devising a training strategy for ADHD and ADRs detection by teachers, it is important to account for the different therapeutic groups that may be prescribed by child psychiatrists when dealing with an ADHD child. The myriad of possible combinations will make it a challenge. However, the ADRs reported in this study are similar to teacher and parent reporting of ADRs found in the literature [27], suggesting that teachers may indeed have a role as an early warning/detector of ADRs. Following the example of other studies published in the literature with other professionals as subjects, new studies could collect ADRs reports in a structured form and duration of symptoms [27]. This allows maximizing the collection of ADRs information, while avoiding confusion between different ADRs.

Teachers’ role in detection and notifying ADRs is intimately related to their relation with parents. In our study, every teacher that found ADRs reported it to the parents. It is understandable that teachers would avoid reporting and ADRs directly to some surveillance system without prior notification of a parent or health professional. As such, and as our results suggest, the focus of training in ADRs detection for teachers should also include communication skills to contact with health professionals after parental consent. An information system that provides the foundation for this communication should be developed. Nevertheless, and assuming that all the reports would be done with prior parent consent, more research is needed on the ethical implications and practicality of having teachers reporting ADRs directly to a health professional or a pharmacovigilance system. Our results show that the National Pharmacovigilance System (NPS) is not sufficiently disseminated amongst the society. The Portuguese NPS allows notifications both from health professionals and from the general population. However, the majority of our respondents reported to be unaware of the existence of the NPS or of the process of ADRs notification. In fact, the surveyed teachers had never reported ADRs detected in their students to the NPS. As for the reason why this report was not made to the NPS, 72% responded that they do not know of its existence or do not know the process by which the report is made. Thus, the need for disseminating information about the NPS and how it works is evident, since teachers are aware of possible adverse reactions that may arise from taking medication and have even detected them, but they only reported them to the parents or the student’s physician, being a passive actor in the spontaneous reporting system. The communication of the reactions observed by teachers to the NPS would also contribute to maintain medication safety and public health.

It seems clear that teachers can notice when a change in behavior occurs, but without proper training and knowledge, they will not know which medicine is causing the ADRs in question. We suggest that community pharmacists could play an active role in this context. Community pharmacists have developed a wide range of services and are the most accessible and available health professional [28,29]. Besides being naturally placed to perceive medications efficacy [30], pharmacists can provide training about medication issues to teachers and parents alike, in or out of the school setting. In doing that, they could be the point of contact with the National Pharmacovigilance System for a multidisciplinary team dealing with a child with ADHD.

Also of importance is the role of new technologies to increase learning capabilities that may assist teachers in dealing with students with ADHD [31]. In this way, a non-pharmacological approach that enhances students performance may help teachers to feel more in control of the classes and to better manage these patients, allowing for increased awareness of therapeutic effectiveness and early detection of adverse drug reactions.

### Limitations

Due to the timing and way the online survey was disseminated and being aware of the low number of respondents in a universe of possible thousands of answers, one must be careful in interpreting and extrapolating these results. Nevertheless, as they match the socio-demographics of Lisbon teachers’ population, the results here presented may represent a large majority of teachers perceptions on ADHD and ADRs detection.

## 5. Conclusions

Our results support the perception that more experienced teachers can better detect and manage children with ADHD. The identification and characterization of the ADRs related to medications for ADHD need to be improved. Thus, teachers need more training to deal with children bearing ADHD, starting at the early stages of their careers and supported by peers. This training should include more topics on drug therapy and associated side-effects. Teachers’ knowledge of a pharmacovigilance system is non-existent, reinforcing the need to present the system to teachers and parents alike. Here, we consider that community pharmacists, as the dispenser of the drug therapy and most accessible health professional, may be well positioned to assume the role of trainer and ADRs reporter in the team dealing with an ADHD child.

## Data Availability

The data presented in this study are available on request from the corresponding author. The data are not publicly available due to privacy restrictions.
